# A Smart Kitchen for Ambient Assisted Living

**DOI:** 10.3390/s140101629

**Published:** 2014-01-17

**Authors:** Rubén Blasco, Álvaro Marco, Roberto Casas, Diego Cirujano, Richard Picking

**Affiliations:** 1 Instituto Universitario de Investigación en Ingeniería de Aragón, Universidad de Zaragoza, C\Mariano Esquillor s/n, Zaragoza 50018, Spain; E-Mails: amarco@unizar.es (Á.M.); rcasas@unizar.es (R.C.); dcirujano@humanopenware.com (D.C.); 2 Institute for Arts, Science and Technology, Glyndŵr University, Plas Coch Campus, Mold Road, Wrexham LL11 2AW, UK; E-Mail: r.picking@glyndwr.ac.uk

**Keywords:** ambient assisted living, ambient intelligence, smart homes, context and user awareness, distributed sensor networks, OSGi

## Abstract

The kitchen environment is one of the scenarios in the home where users can benefit from Ambient Assisted Living (AAL) applications. Moreover, it is the place where old people suffer from most domestic injuries. This paper presents a novel design, implementation and assessment of a *Smart Kitchen* which provides Ambient Assisted Living services; a smart environment that increases elderly and disabled people's autonomy in their kitchen-related activities through context and user awareness, appropriate user interaction and artificial intelligence. It is based on a modular architecture which integrates a wide variety of home technology (household appliances, sensors, user interfaces, *etc.*) and associated communication standards and media (power line, radio frequency, infrared and cabled). Its software architecture is based on the Open Services Gateway initiative (OSGi), which allows building a complex system composed of small modules, each one providing the specific functionalities required, and can be easily scaled to meet our needs. The system has been evaluated by a large number of real users (63) and carers (31) in two living labs in Spain and UK. Results show a large potential of system functionalities combined with good usability and physical, sensory and cognitive accessibility.

## 1. Introduction

The ageing of our populations is a well-known problem in developed countries. European Union population projections are alarming; the ratio of people aged 65 years or over will increase from 17.1% to 30.0% in 2060 (from 84.6 million in 2008 to 151.5 million people in 2060) [1]. Similar figures are found in the USA, where elderly people will represent 20.2% of the population in 2050, or in Japan, with 39.6% [2,3].

Elderly people may suffer several physical and/or cognitive impairments which increase with the passing of years. Old age affects sensing, information processing capability, reduces speed and increases timing of precise movements, *etc.* All these issues increase difficulties of comprehension of complex scenarios which may require multi-tasking or keeping attention over long periods of time. As a consequence, elderly people progressively lose the capability to perform autonomously their daily activities. Thus, household appliances, instead of fostering independent living, become a burden that adds to ageing limitations.

In addition to that, older people are one of the groups of the population most vulnerable to accidents, particularly at home [4]. Most domestic injuries are related to working in the kitchen: kitchen tools, cutlery and household appliances are the most dangerous utensils. As a consequence of these accidents, older people lose confidence in their capabilities, decreasing their self-esteem and consequently, in many cases deciding to move to a nursing home.

In this paper we describe the system built in the European project called Easy Line+ [5]. This project had the objective of increasing elderly and disabled people's autonomy in carrying out their everyday activities in the kitchen. The kitchen is the focus because it is where many activities that are key for autonomy are performed: preparing food, storing provisions, doing the laundry *etc.* Moreover, as evidenced in following section and regardless of its importance, little research has focused in this part of the house to promote independent life.

The remainder of the paper is organized as follows: Section 2 discusses the related work in the field; Sections 3 and 4 describe the system design and architecture, respectively, Section 5 presents the results of the system evaluation accomplished with the target population, and Section 6 concludes the paper.

## 2. Related Work

Ambient Intelligence (AmI) can be defined as a “sensitive and adaptive electronic environment that responds to the actions of the persons and object and cater for their needs” [6]. That is, an intelligent system, customizable, able to be aware of the context, adaptive and anticipatory. This approach includes the entire environment, taking into account each individual object, associating its interaction with humans.

AAL uses the AmI as the essential tool to provide integral solutions for supporting the person in his/her independent living in different contexts: dwellings, transport, workplaces, *etc.* The European Ambient Assisted Living Innovation Alliance (The AAliance) proposes three macro scenarios for AAL development [7]: AAL4persons, with the objective of “Ageing well for the person”; AAL in the community, which is focused on applications in improving the social inclusion of elderly people, their communications and their participation in the community; and AAL@work, which is focused on application supporting elderly and people with disabilities at work.

Therefore, as O'Grady *et al.* pose [8], an “Ambient Assisted Living (AAL) is advocated as technological solutions that will enable the elderly population maintain their independence for a longer time than would otherwise be the case”. To achieve this goal, the AAL should be aware of context, including the person, providing help when needed, detecting abnormal situations and acting accordingly.

We can find several works focused in deploy AAL with different purposes, for example the MONAmI project selects suites of technological services to support people at risk of exclusion and loss of autonomy [9] or the Necessity system proposed by Muñoz *et al.* which offers a system to represent and validate alerts in a domestic environment [10].

Focusing on activity recognition in the kitchen, Lei *et al.* [11], show a system based only in a RGB-D camera (modern depth cameras that provide synchronized color and depth information at high frame rates) which identifies activity and tools used (between a selected group of 35 objects and 25 actions). The system is capable of identifying objects with an accuracy of 60% and activities with an accuracy of 82%. Suryadevara and Mukhopadhyay [12] developed and tested an intelligent home monitoring system based on a wireless sensors network (no camera or vision sensors) to monitor and evaluate the well-being of the elderly.

On the other hand, several AmI have been developed to help and guide the user in different activities performed in the kitchen. Ficocelli and Goldie [13] present an assistive kitchen with speech communication and an automated cabinet system to ease storing and retrieving items and to obtain recipes for meal preparation. We can also find systems that guide the user to have a healthier diet. The Smart Kitchen proposed by Chen *et al.* [14] offers information in real time about the calories, nutritional value and their balance (nutritional & calories). The system detects when a new ingredient appears and then asks the user about the name of the ingredient in order to update the information of the screens.

The user interface is essential in all these processes involving elderly and technology. The i2Home project tries to make appliances and devices easier to understand for people with mild cognitive impairment and the elderly using a new mainstream user interface standard: the Universal Remote Console (URC) [15]. Although this project is not focused in the kitchen, it creates support services integrating several technologies and devices: appliances (hood, oven, fridge, freezer, dishwasher and air conditioning), touch screen, RFID antenna which implement sensitive surfaces for products equipped with smart labels and lighting equipment [16]. Schwartze *et al.* [17] present their work in graphical interfaces for Smart Environments with the “4-star Cooking Assistant” application which proves the capability of their system to dynamically adapt a graphical user interface to the current context of use.

Augmented reality techniques have been also tested in the kitchen. The work of Bonanni *et al.* [18] presents a conventional kitchen with the projection of information onto objects and surfaces to help people cook more safely and easily. Also, several objects could be easily integrated in an AmI, such as the Intelligent Spoon [19] which is able to measure the temperature, acidity, salinity and thickness of food or the Chameleon Mug [20] which determines the temperature and sugar level of liquid and, even, the state of the milk.

## 3. System Description

To our best knowledge, the architecture, implementation and end-user evaluation of the AmI kitchen proposed is new in the field, providing four main functionalities within the kitchen scenario: (i) it facilitates the use of household appliances; (ii) it provides useful information and warnings about the use of household appliances; (iii) it detects emergency situations and takes corrective actions when needed; and (iv) it analyzes all the data gathered to extract relevant information that could be useful for the user's carers and/or relatives in order to evaluate the person's quality of life.

Two main principles guided the system design: resistance to obsolescence and ability to interoperate with existing systems in the field (such as white goods, sensors or RFID from different manufacturers). It is evident that “intelligence” and user interfaces need to be integrated in the kitchen; nevertheless, this doesn't mean that white goods have to be more intelligent or incorporate new adapted interfaces. This would increase their unitary price, complicate their installation (adapting the functionality to the user's particular case requires configuration) and consequently hinder the market penetration. Thus, instead of having new smart appliances with accessible interfaces, a central intelligence entity has been developed, which we call the e-Servant. It is conceived as a set of interchangeable blocks with defined communication interfaces to grant interoperability among existing systems. This way, any electrical appliance with communication capability can be integrated. As a result, the development and stability of the appliances eases (they don't change their current way of functioning, but they just need to add communication to inform about their status and execute actions). Additionally, if new household appliances, sensing systems, user interfaces, *etc.* appear, as long as they have a communication interface to interoperate with them, they can be integrated in the platform.

**Figure 1.**
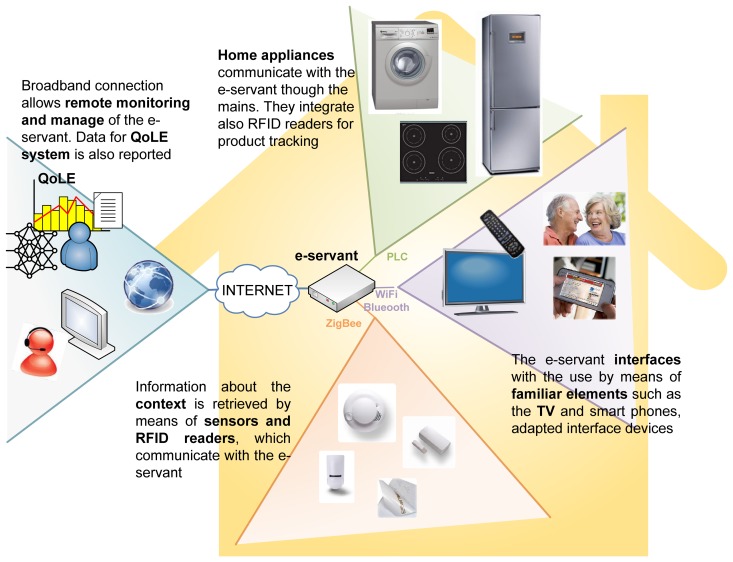
Smart Kitchen system.

In order to validate the system with the most exigent conditions, besides different household appliances, sensors and multimodal user interfaces, we integrated as much communication media as possible: Power Line Communication (PLC) for the white goods, RFID for item identification, ZigBee as wireless sensor network, infrared for the remote control, Bluetooth for audio streaming and Ethernet (WiFi and cable) for cloud and user interaction. Figure 1 illustrates the proposed system.

### 3.1. Context Interaction

In any AmI application, the physical context information is essential because these data are the input for the logical rules and decision processes in order to create services. With respect to the kitchen scenario, white goods, user interfaces and sensors are the main information sources from the context.

As already mentioned, electrical appliances should be able to report their status and be handled by the system or by the user through the built-in interface. In the implementation of the system, we use a range of commercial white goods that use PLC, but they are also required item identification capabilities to provide smart services. In that sense, Radio Frequency Identification (RFID) technology comes to the rescue. RFID technology is increasingly more integrated in our daily lives. It is expected that many goods will store identification informing about the expiry date (food), washing temperature (garments), dosage (medicines), *etc.*

As conventional white goods are not able to provide all the information needed, RFID readers with ZigBee communication have been incorporated in the appliances in order to enhance the capacities of the fridge and washing machine. Also, as food is not only stored in the fridge and it is not feasible to put readers in every cupboard, a stand-alone RFID reader was set in the worktop to gather information about any specific item. A textile patching label including RFID chip and metalized thread technology that is attached to garments and can work for long periods of time, supporting several washing and ironing cycles, was also developed.

Standard sensors (gas, fire, smoke, flooding) are commonly used inside kitchens to detect emergency situations and automatically take evasive action, such as actuating the closing of the mains supply. Other sensors have also been considered that are not so commonly used in this scenario: magnetic sensors—to detect when the user opens/closes a cupboard or drawer—light sensors—to detect when the user forgets that the lights are still on—presence sensors—to detect when the user enters the kitchen—*etc.* All these sensors use the ZigBee wireless standard as it is one of the most appropriate for home control and automation and easily allows adding new devices to the system.

Other sources of context information are user interfaces. Human-Machine Interface (HMI) devices must be simple to use and must be available for any kind of user, having the capability to change the interface according to the user profile. They must have a consistent communications interface, without the need for a powerful processor or large storage capacity.

**Figure 2.**
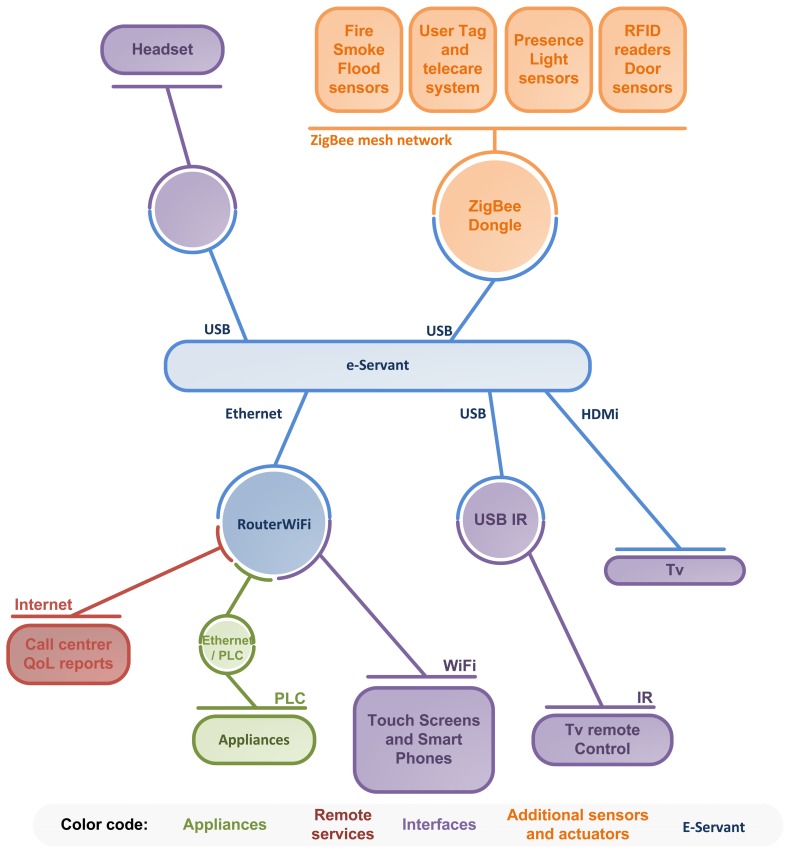
Communication diagram for context interaction in the Smart Kitchen.

There are many different types of clients that can be used to manage the system: mobile devices (smart phones, wearable devices, ultra-mobile PCs, touch screens, *etc.*) which allow the user to carry them around the house and enabling them to monitor the house appliances wherever he or she is; fixed devices (tactile computers or digital TV), which will be used as a centralized control; and finally embedded devices which may be control panels attached to each of the current appliances. Figure 2 shows how context interaction elements are connected to the e-Servant.

### 3.2. E-Servant

In the architecture proposed, the system intelligence is provided by the e-Servant. This device is an embedded computer that centralizes the communication with the kitchen appliances, sensors and interfaces as well as behaving as a gateway to the outside world. The e-Servant is defined as the central hub of the whole system that, being aware of the context and user, enhances its intelligence. It is also a learning system, which detects and compensates the behavior, habit changes and loss of abilities of the user. With or without user cooperation, it facilitates the use of the appliances, adapting the system to the capabilities of the users.

For each user interaction, the e-Servant knows how to help the user following operational rules which take into consideration the user capabilities and environmental context. Also, the e-Servant is checking continuously the state of the kitchen appliances, providing warnings through its user interfaces (TV, tactile screen and smart phones) if there is any problem or event to be notified (e.g., the fridge door is open or the cooking has finished).

The e-Servant is also able to detect emergency situations in the kitchen (merging the information provided by the sensors described in subsection 3.1) and automatically takes corrective actions if the user does not respond.

Finally, the e-Servant also manages records with the relevant events that have occurred in the kitchen gathered from the context (sensors, kitchen appliances) and user interaction. This data is processed and analyzed using artificial intelligence methods in order to extract findings about the cognitive level of the person that could be useful to the carers and/or relatives. This information is used to create an expert service called Quality of Life Evaluation (QoLE) with two main outputs:

- A detailed report is automatically produced and forwarded via e-mail to the user's carers or relatives. This report contains the habits of the user in four areas of daily living: food management, cooking, doing laundry and other activities useful to follow the evolution of the person in the kitchen.
- The support level of the system is defined in the user profile that should be completed and updated carefully by the career or the relatives that know the user. After analyzing all the information available, the e-Servant periodically suggests to the career or relative to increase or decrease the amount of support that the system is providing the user in the kitchen.

The area outside the house in Figure 1 shows the services that the e-Servant provides through internet connectivity. It sends information about the user to carers and relatives, establishes a connection to the call center or the emergency center when needed, allows remote maintenance and updates to the system, *etc.* Also, due to the flexible software architecture implemented, it easily allows addition of new services to the system such as on-line shopping. In the end, the e-Servant is the coordinator with whom all the other subsystems communicate using the most appropriate way of communication: PLC, ZigBee, WiFi, Ethernet, *etc.*

## 4. Software Architecture of the Smart Kitchen

Consumer electronics manufacturers, as “smart homes' providers”, are influenced by consumer demand for easy-to-use, stable and enduring technologies but, at the same time, they require new services and features incorporated therein [21]. This phenomenon implies that providers must be able to develop these new features quickly to avoid losing market share, while ensuring the products continue operating as before. Developing monolithic software applications for such systems will prove to be expensive and, in the medium-term, unsustainable. Thus, nowadays it is usual to find designs based in Service Oriented Architectures (SOA) that permit the development of modular, and easy to update software applications [22,23].

A SOA technology being adopted massively in this kind of applications is the Open Services Gateway initiative (OSGi) [24], as demonstrated by initiatives such as the European project UniversAAL [25] and the Ambient Assisted Living Open Association (AALOA) [26]. The UniversAAL project brings together many of the leading European players who have participated in a number of European projects of the sixth and seventh framework programmes, in which were posed architectures to support AAL applications, and they intend to propose an architecture and reference implementation for AAL scenarios, where OSGi is used as middleware.

OSGi defines a framework where pieces of code are organized into bundles that can be managed separately. OSGi bundles are agents which might be dedicated to specialized tasks, such as handling a serial port, providing a command line interface, collecting, aggregating and analyzing data, *etc.* These bundles communicate and interact with each other by means of services which are published within the framework, and each bundle can acquire and utilize them.

**Figure 3.**
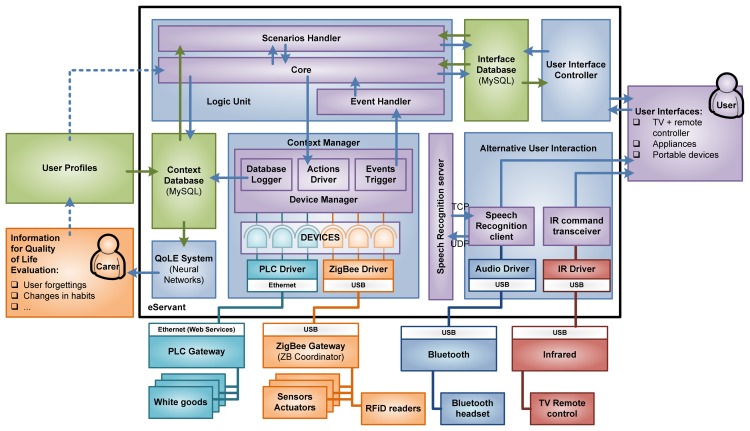
Software architecture of the e-Servant.

The main strength of OSGi is that the framework manages these bundles dynamically, allowing them to be upgraded without terminating the full application, as well as enabling the availability of the services to other bundles depending on the situation. That allows providing new features and capabilities by adding new services which may use existing ones, but keeping current features unaltered. Therefore, OSGi has been chosen as the backbone of the e-Servant in order to enhance its capabilities, and decreasing the cost of maintenance in a future commercial product (easy update of software packets, addition of new sensors or appliances, *etc.*). Our system consists of a number of components, as can be seen in Figure 3, which are detailed next.

### 4.1. Context Manager

The Context Manager (CM) plays the role of interface between the virtual and the real world. The information about the status of the appliances, product inventory, user actions or any other event is gathered by the CM and sent to the Logic Unit (LU) which will decide whichever operation must be performed (control the appliances, generate remote alarm calls via care-phone, *etc.*). The CM is the agent responsible for retrieving that information, processing and presenting it in a structured way, and it is organized in three levels: drivers, devices and devices management.

#### 4.1.1. Driver

The lowest layer of the context manager is the driver layer. In this level, communication with physical devices and transport services are developed. The tasks carried out by this layer are:

- Physical channel establishment. The driver layer has the responsibility of creating and opening communication ports with the network gateways. Automatic identification of ports is also performed if possible (*i.e.*, serial port scan for the connected gateway in the ZigBee Network sensors set).
- Device enumeration and network support. Once the communication channel is working, sensor network management is carried out by the network driver, which deals with network support operations, and performs enumeration and registration of the physical devices integrated in the network infrastructure.
- Device instantiation and messaging service. Devices recognized by the driver in the network are instantiated by the driver layer and presented to the context manager, allowing exchange of information between them and their software representation.

Two drivers have been implemented into the context manager architecture for communicating with physical devices:

- PLC driver to communicate with kitchen appliances. The PLC driver is connected to a PLC gateway through Ethernet, using web services. The driver checks that the gateway is running, and registers the devices (appliances). For each device, a PLC Device is instantiated and presented to the context manager.
- ZigBee driver to communicate with sensors, actuators, RFID readers and care-phone. The ZigBee driver establishes the connection with the ZigBee gateway, which also acts as the coordinator of the network, through a USB (serial) port. The driver checks if the network is created and, if so, gets the devices connected in the network. For each device in the network, a ZigBee Device is instantiated and presented to the context manager.

#### 4.1.2. Devices

Each of the above described drivers instantiate object devices related to the physical devices connected, or more precisely, controllable by means of the respective driver. These objects correspond to OSGi services, which have an independent identity within the framework following the OSGI4AmI Ontology [27,28].

Device representation allows separating device functionality from the underlying technology and the transport layer. Each one holds data members, properties and methods which model the behavior of the physical devices they represent. Activation or access to these methods implies the communication with the physical devices through the base driver that has instantiated the device (therefore, the base driver must be active to enable that communication).

Devices maintain a link with the driver which has instantiated them, and OSGi provides the mechanism to dynamically modify this link if the base driver disappears (for example, if a network gateway becomes unavailable). However, the technology gateways involved in the application scenario suggest uninterruptible availability, and its disappearance will lead to devices not being reachable at all, so this link is established when the device is first created.

#### 4.1.3. Device Manager

The lower layers of the CM gather existing physical devices and present them in a structured way. The Device Manager is responsible for manipulating and aggregating information from the devices and effectively offering the context awareness to the upper layers. The main tasks performed by the Device Manager are:

- Database logging. Gathered information about sensors together with the derived information from the context aggregation is logged into the Context Database, which will be used by the LU and Quality of Life Evaluation (QoLE) services.
- Action driving. Through this module, The CM allows the LU to act over the physical devices.
- Event triggering. Received information from the sensors is stored in the context database, but some external event may require an immediate response by the e-Servant. This module notifies the LU that something is happening.

Tasks carried out at this level are achieved by means of several OSGi bundles, which recruit the required devices and perform necessary actions with them, publishing themselves as services and being available to be used by any other service. Without detriment to the preceding procedure, instantiated devices are also available to be directly accessed if needed.

**Figure 4.**
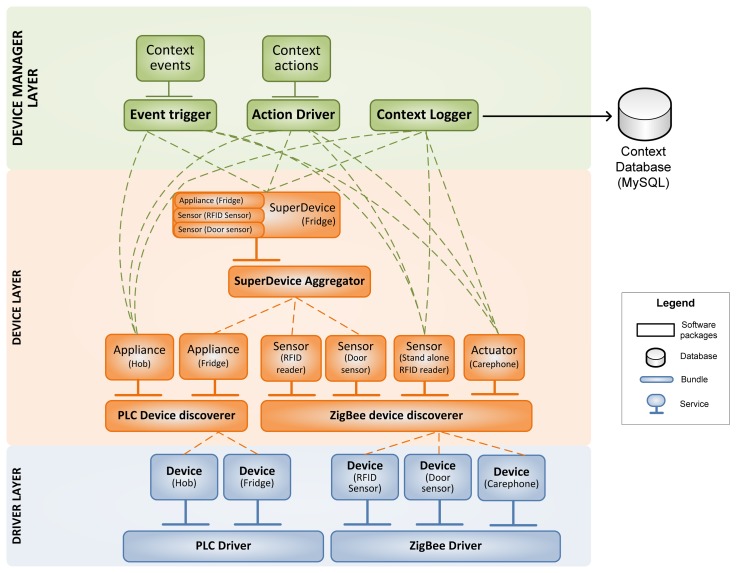
Architecture detail of the Context Manager.

Figure 4 shows an example of how the CM layers work. At the Devices Layer, there are two bundles, PLC Device Discoverer and ZigBee Device Discoverer, looking for devices offered by drivers on the Driver Layer and publishing them also as services. Above them, the SuperDevice Aggregator bundle is looking for devices which form part of a *superdevice* (a device which aggregates functionalities from several simple devices), in this case the Fridge. Finally the Device Manager layer groups the information of the context and isolates upper layers of the CM.

### 4.2. Logic Unit

The Logic Unit is the “brain” of the e-Servant, responsible for three main duties performed by three different services: to process all the information provided by the context manager (Event Handler); to reason through that information and deciding actions in order to support the user (Core) and actuate over CM devices if required; and to cooperate with the User Controller Interface (UIC) to manage the interaction with the user from a logical perspective (Scenarios Handler).

**Figure 5.**
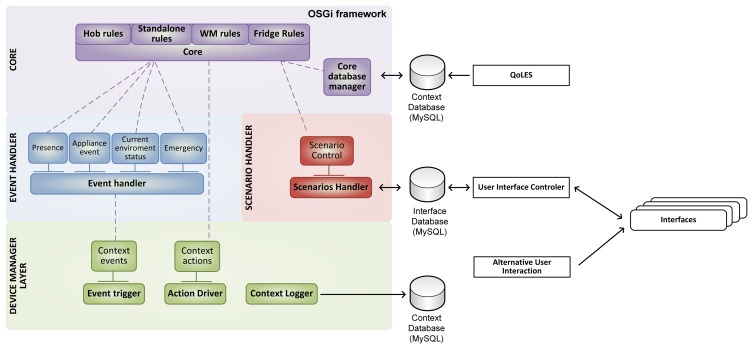
Architecture detail of the Logic Unit.

These blocks can be seen in detail in Figure 5. The LU continuously analyzes the information coming from the CM to detect what is happening in the kitchen. When it detects a situation where the user might need guidance, help or information, it starts a communication with the user; this interaction is called a user-scenario. The LU starts and stops completed user-scenarios through the interface database, and execution of the user-scenario is done by the UIC—that manages the different user interfaces—notifying the result of the interaction back to the LU through the interface database. The LU also stores relevant high level events related to the user behavior that will be used by the QoLE service.

It is important to remark that the e-Servant's main job is not to replace the user in his daily activities, but supervising if the user is doing them correctly, and providing help when needed. The support level that the system provides is directly related to the user profile.

The possible problems, oversights or mistakes the user might have are recorded in the Context Database to be analyzed by the QoLE System. Its conclusions are forwarded to carers or relatives so they could update the user profile if considered; changes in the user profile are always done with human supervision. Next, we explain in detail the sub-systems of the LU.

#### 4.2.1. Event Handler

The Event handler is a listener of the events generated by the CM. When the system is turned on, the Event Handler waits for new devices. As soon as a device appears, the Event Handler registers itself as its listener. From a logical perspective, the CM offers virtual objects which are representations of the devices. These objects permit the LU to know the status of sensors, appliances, *etc.* and control them through the methods that they provide.

#### 4.2.2. Scenario Handler

The Scenario Handler provides control methods (start, stop, pause) to manage the execution of user-scenarios that are run by the UIC. A bidirectional communication is established through the Interface Database which is polled in a timely fashion (every second) in order to know which user-scenarios are running, waiting or interrupted. Thus, similar to the events reported by the CM, the Scenario Handler reports to the Core any event happening for each user-scenario running.

#### 4.2.3. Core

The Core is a service that takes the decisions that rule the system. These rules are deterministic because, for security reasons, it is mandatory to know what the e-Servant will do in every situation. Logic is driven by events arriving from the context (Event Handler) or from the user interaction (Scenario Handler) indicating that the kitchen situation has changed. Then, the Core analyses the new situation of the kitchen considering the user profile, status of the kitchen appliances, user interfaces and sensors, *etc.* and decides if it is necessary to start any action with the user or with the white goods:

- Actuate over a device. The Core can turn on/off, configure and command the white goods present in the kitchen; send a notification to a technical support center when breakdown is detected; forward a warning to a call center in order to inform about an emergency.
- Update the status of the appliance in the user-interface. When the status of an appliance, food or clothing inventory *etc.* changes, the Core updates the information in the user interfaces.
- Interact with the user launching a user-scenario. If interaction with the user is needed, the Core will update the interface database to indicate to the UIC which user-scenario should be run.
- Save information into the context database. If there is relevant information, the Core will save it for its subsequent analysis by the QoLE System.

The concatenation of these actions for each specific situation constitutes a rule. Table 1 shows some of the rules defined for specific situations determining the behavior of the LU. Depending on the user profile, each rule is defined differently (even being possible to disable if needed). Also, because of its modular design, it is very easy to add new rules and user-scenarios in order to enhance system functionality without making changes to the architecture.

### 4.3. User's Profile

While interacting with the e-Servant, it is possible to adjust how and what information is sent to the user. Customizing the physical parameters of the output channels of the system, such as volume, pitch, contrast, *etc.*, it is possible to control how the information is send to the user. Also, the e-Servant can increase the help level which is offered to the user as well as the information shown to him/her. This customization is done thanks to the user profile.

Users' profiles have been studied using the “persona” concept [29]. This idea, developed by Alan Copper in his book “*The inmates are running the asylum*” defines personas as “Personas are not real people, but they represent them throughout the design process. They are hypothetical archetypes of actual users” [30]. In this case, ten personas have been defined based on European statistics randomly assigning age, education, work, family situation, impairments and technology background. User profiles aim to consider cognitive and sensorial capabilities of the person within the following categories:

- User level has four different grades: not possible (0) indicates that the user is not able to use the system; of course it could be a temporary situation. Easy (1), standard (2) and expert (3), indicates the understanding that the user has of the system. This understanding can be due to different reasons: e.g., knowing all the system's features, having memory losses, *etc.* From the LU's point of view, this is the most relevant parameter as it is related with the cognitive capabilities and the technological skill of the person. This parameter determines the interaction with the user (number of options, complexity of menus, *etc.*).
- Interface makes reference to how the system will show the information to the user: using icons (0) text (1) or both (2). Besides the user preferences, this also has implicit information about the user's cognitive level.
- Audio: Inside audio category, three sub-categories are included: Volume, pitch and voice control. The first two can help people with aural disabilities to hear the HMI. Voice control indicates if the user would control the system via voice commands. Of course, this would be helpful for people with visual disabilities, but not only these. As voice is the most natural way of communicating (compared with remote controls, keyboards, tactile interfaces, *etc.*), voice control would be helpful for those people with reduced cognitive capacities or low technological skills.
- Display: it includes common adjustment controls in screens: contrast, brightness and colour settings (physical parameters). These characteristics, besides adapting to the ambient light and user preferences, together with magnification might help people with visual impairments to interact with the display.
  The user profile has been validated setting a different user configuration for each persona.

### 4.4. User Interface Controller

User-scenarios guide and help the user in his/her interaction with the smart ambient environment. Each user-scenario embeds the visual and aural information necessary to maintain an interaction with the user.

As seen in Figure 3, the LU launches user-scenarios using the Interface Database. The UIC periodically polls the database refreshing the information served to the user interfaces that will play the user-scenarios. Then it manages the dialogs with the user, writing the user's answers in the Interfaces Database to be parsed back by the Scenarios Handler in the LU.

**Figure 6.**
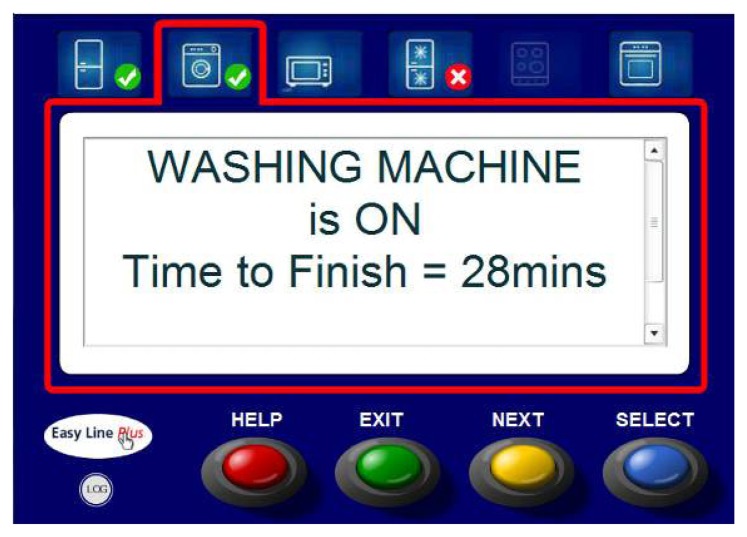
User interface screen showing information about the washing machine status.

To ensure that the user accesses the same interface no matter which client he/she uses, the UIC serves the information to the user interfaces through an embedded http server. Each user interface employs a small Adobe Flash application that retrieves the information from the UIC and displays the user interface, as can be seen in Figure 6.

### 4.5. Alternative User Interaction

Despite the accessibility of the interfaces served by the UIC, an Alternative User Interaction (AUI) module has been implemented to provide additional methods of interaction.

Speech is one of the most natural ways of commanding user interfaces, but its implementation entails the problem of limitations and interoperability of the platform used. To overcome that, a client—server schema has been proposed, and a Speech Recognition Server (SRS) has been designed to allow devices with limited resources to use a speech recognition engine. On the client side—the user interface—it only needs a small application called the Speech Recognition Client (SRC) in order to support voice control over the system. This client gets the audio stream from the embedded microphone, packetizes and forwards it through a socket to the SRS. The server analyses the audio stream and replies to the client with the identified command. In our case, we implemented it using the Operating System's recognition engine installed where the e-Servant is running, but its model would allow easy replication using any speech recognition engine in the cloud.

Another interface widely extended is the TV remote controls, and elderly people are usually familiarized with it. A commercial IR to USB transceiver [31] has been used in order to integrate this technology in the e-Servant. IR commands are parsed by the IR command transceiver module and converted in understandable stimuli for the e-Servant interface.

### 4.6. Quality of Life Evaluation System

The Quality of Life Evaluation System is a service that periodically (a period configurable between 1 and 3 months) analyses the context database looking for changes in the user washing, shopping and cooking habits which could be relevant in order to detect a loss of physical, cognitive or sensorial capabilities; for example, if the user starts going to the fridge at night (might indicate insomnia) or if s/he is doing the laundry less and less often (might indicate that he/she is wearing dirty clothes). This allows performing an indirect evaluation of the quality of life of the user through the measurement of its capabilities and habits in his/her daily tasks in the kitchen. It is designed for the use of non-technical people, and it produces an understandable report aiming to provide objective information about the everyday life of the user.

This service is intended as a tool for social workers to complement the information they typically use (surveys and personal interviews) to assess the user's quality of life. Personal interviews often are influenced by many factors such as empathy between the social worker and the elderly person (that may modify the person's mood and consequently produce a bias) and also dependency on the person's mood variation through the day, week, *etc.* (observation in an interview is an isolated event in time which may produce a bias). Thus, the QoLE service increases objectivity, data reliability and the amount of data gathered to work as a ‘Decision Support System' for professional carers or relatives. It can expose information which, for example, could be useful to set the level of support the system provides.

### 4.7. Use Case Example

**Figure 7.**
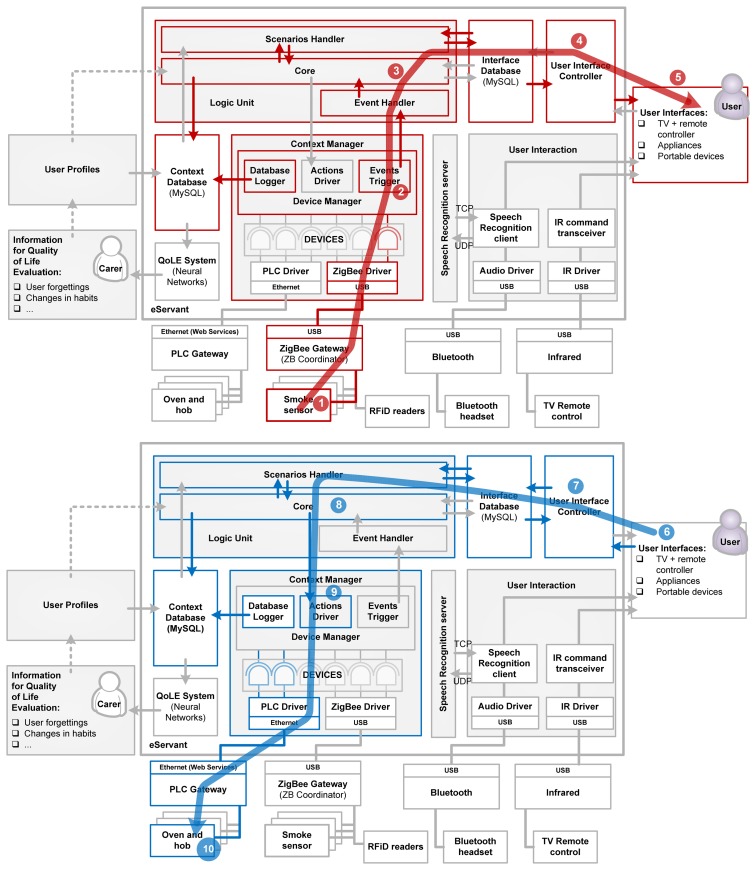
Use case: smoke sensors notify the system that there is smoke in the kitchen, oven and hob are on but nobody is in the kitchen.

To illustrate the interaction of the various blocks of the architecture, consider the use case drawn in Figure 7, in the event of smoke detection. The ZigBee smoke sensor (1) warns to the CM (2) that there is smoke in the kitchen. LU (3) is notified and decides to launch a user-scenario to warn to the user. UIC (4) commands the interfaces (5) in order to warn the user about the situation. After a timeout, the interfaces (6) notify to the UIC (7) that the user does not interact with them and the LU (3) decides to turn off the PLC hob and the oven (10) through the CM (9).

## 5. System Evaluation

When technology is evaluated, it is necessary to consider a range of social, technological, institutional and personal factors [32]. Additionally, legal and ethical aspects have mandatory consideration when users are elderly or disabled [33]. In this context, there is an interdisciplinary crossing between the technical development of the innovation and its implementation in intervention situations with social assistance which could be considered itself as trans-disciplinary [34].

Literature address this situation from different approaches: about user satisfaction [35], about its psycho-social impact [36], about the person, his environment, the technology and the impact of the period training [37], about the functional independence [38], or how they affect the quality of life [39,40]. Regarding the evaluation of the Assistive Technology (AT), we can find that the use of interaction models in the assessment is quite usual [41]. These models are used mainly for AT outcomes research highlighting the next Human Activity Assistive Technology (HAAT), Matching Person and Technology (MPT) and the ICF [42–44]. In the end, it is usual to design a specific “tool” in order to obtain evidences about if a particular technological innovation responds or not to the purpose for which it is designed.

Although technology assessment has traditionally followed quantitative techniques based on the accepted convention of their rigor, the prevailing reality shows that qualitative assessments can be equally rigorous. In fact, the prevailing trend tends to combine both [45–51], minimizing the prejudices of each of these methodologies through the contrast processes. This is the philosophy that has been followed in the design of the system assessment: to combine qualitative tools such as observation or interview with quantitative tools such as surveys and data collection.

### 5.1. Assessment Approach

**Figure 8.**
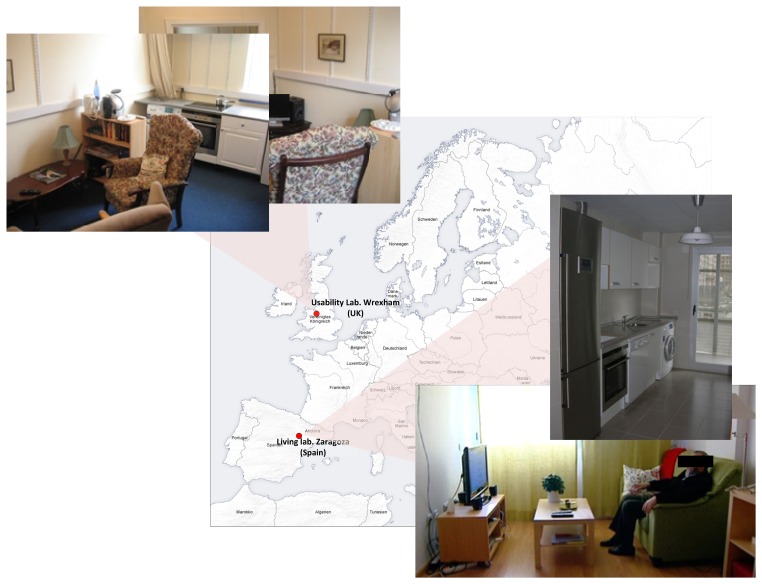
Evaluation places.

The system has been evaluated by 63 end users and 31 formal and informal carers in two living labs placed in Spain and UK (Figure 8). Spanish trials were conducted by the University of Zaragoza, and UK trials were conducted by Glyndŵr University, in Wrexham. Profiles of beneficiaries recruited regarding disabilities, age range and gender are shown in Table 2. Note that disabled people younger than 59 years old have been recruited to increase the ratio of people with disabilities. A relevant limitation that hinders obtaining conclusions about the ability of the system to cope with specific disabilities is that users usually have more than one disability being very difficult to isolate the effects of each one over the system use.

### 5.2. Assessment Methodology

**Figure 9.**
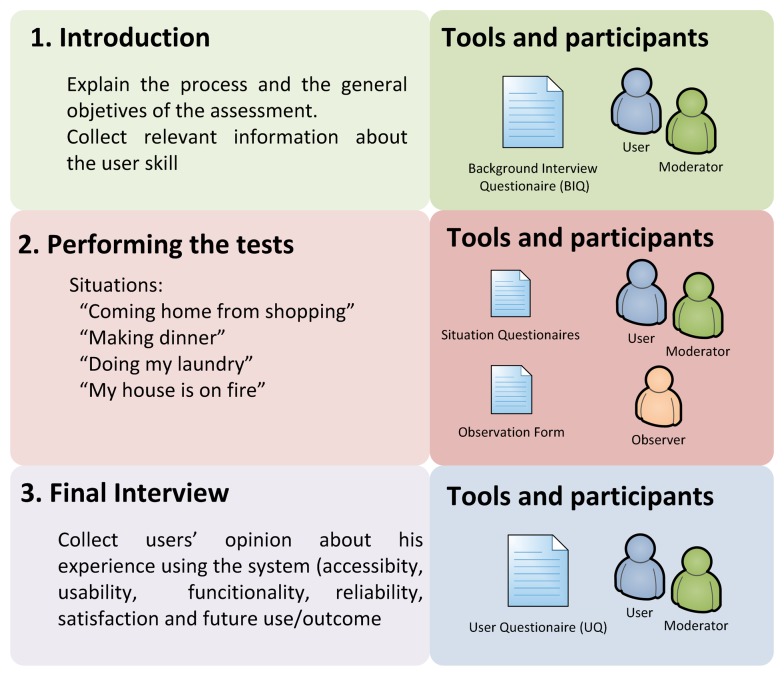
Evaluation process.

Each user evaluates the system through four specific situations after a brief period of training. There are three people participating in the assessment whose roles have been defined as follows: The user is the person who will evaluate the technology, the test moderator who leads the sessions and the test observer who is watching the different situations evaluated without contact with the user, taking notes about the participants' performance and reactions. Each situation has been designed in order to allow evaluation of the main system's functionalities studying the interaction between user and system. Figure 9 summarizes the process.

### 5.3. Evaluation Results

**Figure 10.**
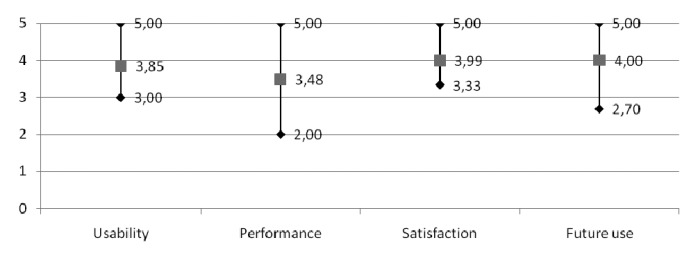
Usability, performance, satisfaction and future use rates.

Evaluation methodology details and full results are available in the public deliverable of the project “7.2. Report with the results of the end users' test” [52]. In summary, we can say that the system has good usability and physical, sensory and cognitive accessibility; 90% of the users that evaluated the system found it accessible; usability has been evaluated with a score of 3.85 out of 5 overall, on a rating scale of 1 (poor) to 5 (excellent). Figure 10 shows the evaluated parameters and their 95% confidence interval.

**Figure 11.**
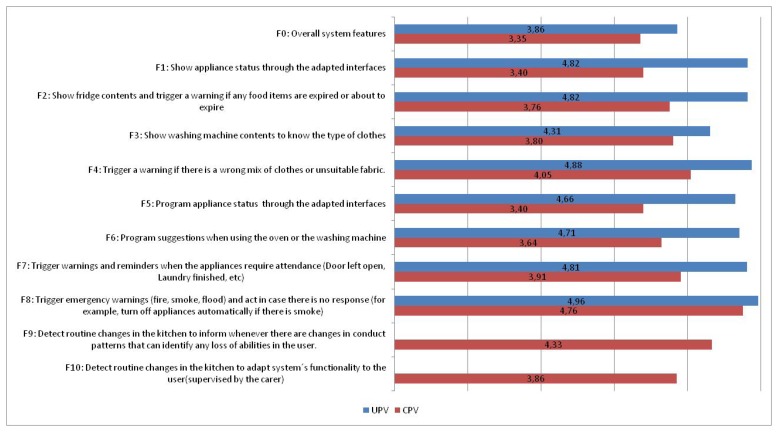
Functionalities of the e-Servant evaluated by the caregivers (Caregivers Point of View, CPV) and for the users (Users Point of View, UPV).

Regarding the functionalities of the system, Figure 11 shows how beneficiaries and carers rate them.

### 5.4. Evaluation Discussion

Assessment of AAL systems with real users is a challenging task for different reasons. A key lesson learnt is the importance of a coherent assessment methodology integrating both qualitative and quantitative instruments (observation, questionnaires, discussion groups, role playing, *etc.*). Evaluation activities should also target end users and caregivers in order to get a complete picture of the reality. Besides obvious ethical and privacy issues, other practical considerations [53] are to provide enough training before testing, and not putting vulnerable people on their own unless it is absolutely necessary, consider bias introduced by the Hawethorne effect, provide a realistic and familiar layout, keep tests short and allow some script flexibility if needed. Additionally, the system must be functional and robust enough to be used by end users. In this sense, the architecture and implementation of multiple systems and communication standards as proposed proved to be technically feasible.

On not yet analyzing specific evaluation results, it is noticeable how caregivers give much less importance than end users to the use of the kitchen (show information/program appliances through adapted user interface); something that *a priori* would help end users using white goods for more time and thus remaining independent longer. Everybody coincides in the importance of alarm detection and handling in case there is no response. And carers also bestow great importance to “Detect routine changes in the kitchen to inform whenever there are changes in patterns of conduct that can identify any loss of abilities in the user” as it can strongly improve the tools that they have to monitor the progress of elderly and disabled people.

Regarding the interfaces, the majority of users felt more comfortable using the television remote control of television as interface, followed by the touch screen which has proven to be quite intuitive. By contrast, the use of voice commands have only been used by those users who really had problems with other interfaces.

The general results of evaluation have been discussed in several workshops with professionals confirming that there are clear indicators that the functionalities of the system have a big potential to support the user in several areas of Activities of Daily Living (ADLs), reducing the dependence level of the person and thereby increasing his/her time of independent life. The system can support the user in the ADLs' areas of carrying out domestic tasks (preparing a meal, doing the shopping and doing laundry/ironing) and making decisions (about domestic tasks). Early detection of changes in routines would also help to monitor quality of life of the user in some aspects and take preventive actions.

## 6. Conclusions

This paper presents the design, development and validation of a smart environment which supports independent living in the kitchen of elderly and disabled people. The system concept and its implementation are innovative, merging many different technologies to build the smart environment: RFID technology, wireless sensor networks, distributed computing, artificial intelligence, *etc.*

The backbone of the system is its modular architecture based on an OSGi framework where different bundles (independent pieces of code) are in charge of providing the required functionalities. It also allows easily adding new devices and functionalities, or replacing some device without interfering the system and minimizing the adaptation effort. For example, by adding new drivers to the Context Manager, the system could support any sensors, devices or white goods with any communication media. In our case, sensors and RFID readers with ZigBee, remote control with infrared, white goods with PLC and Ethernet have been implemented.

Functionalities of the system can be easily expanded by adding rules and user-scenarios to the Logic Unit and User Interface Controller, which may use existing devices or new ones. Even the kitchen scenario can be expanded to the whole house by defining new user-scenarios, without modifying the system architecture, nor the existing rules.

From the user interaction point of view, any IP-enabled device, such as the latest smart phones and tablets, can be used as user interfaces of the system, and it is possible to build alternative interfaces that access directly the Logic Unit without interference to the existing ones. This allows the system to be easily adapted to each person's needs with minimal or no change in the system.

Taking advantage of the information collected with the system, a Quality of Life Evaluation Service has been developed. The QoLE system uses artificial intelligence to detect routine changes in the kitchen and to inform whenever there are changes in behavior patterns. This allows progressive personalization of the system and early intervention of the carers when loss of abilities of the user is identified.

The evaluation with real users and their carers demonstrated that the system developed has the adequate functionalities and user interfaces as it is physically, sensory and cognitively accessible and usable for the elderly and disabled. We have found evidence that this system would prolong the time elderly and disabled people could remain independent in their own homes, thereby positively impacting their quality of life, although longer studies with larger user groups would be needed to confirm this.

## Figures and Tables

**Table 1.** Rules categorized by appliance.

**Appliance**	**Rules**
**Washing machine**	Load incomplete Incompatibility of clothes detected Help to program the washing machine Washing cycle interrupted Washing cycle completed Washing machine is broken down

**Fridge**	There is some food item expired/about to expire The door of the fridge is open The fridge is broken down The user would like to change the settings of the fridge

**Hob and oven**	Danger situation detected (fire, smoke, hob is on and no pan) Hob/oven is broken down

**Standalone RFID reader**	Display information about item Configure an appliance

**Table 2.** Recruited participants' characteristics. (Note that a person could have more than one disability).

**Characteristics**	**Recruited Participants**
**Impairment disability**	
**None**	26
**Visual impairment**	13
**Hearing impairment**	13
**Cognitive impairment**	12
**Motor impairment**	23
**Age range**	
**<59**	11 (8 female)
**60–79**	46 (28 female)
**80+**	7 (4 female)
**Gender**	
**Male**	40
**Female**	23
**Total**	63
